# Supplementary material for: KRT17 promotes triple negative breast cancer through activation of Wnt signaling and γδ T-cells recruitment
Source: Commun Biol. 2026 Mar 26;9:676. doi: 10.1038/s42003-026-09897-0 (PMC13183921; doi:10.1038/s42003-026-09897-0)
Supplement: Supplementary file 1 — Supplementary Information [file 42003_2026_9897_MOESM1_ESM.pdf]

KRT17 promotes triple negative breast cancer through activation of Wnt signaling and $\gamma\delta$ T cells recruitment

Chermakani Panneer Selvam[1,2], Gatha Thacker[1,2], Ukjin Kim[1,2], Youley Tjendra[3], Melinda M Boone[1], Samantha Henry[4], Camila O Dos Santos[4], Rumela Chakrabarti[1*]

[1]Sylvester Comprehensive Cancer Center, University of Miami, Miami, FL 33136, USA.

[2]Department of Surgery, Miller School of Medicine, University of Miami, Miami, FL 33136, USA.

[3]Department of Surgical Pathology, Miller School of Medicine, University of Miami, Miami, FL 33136, USA.

[4]Cold Spring Harbor Laboratory, Cold Spring Harbor, NY 11724, USA.

[*] Corresponding Author

[*] Corresponding Author

Correspondence to:

Rumela Chakrabarti, Ph.D.

Department of Surgery

Miller School of Medicine

University of Miami

Miami, FL 33136, USA.

Phone: 305-243-6545

E mail: rxc1335@miami.edu

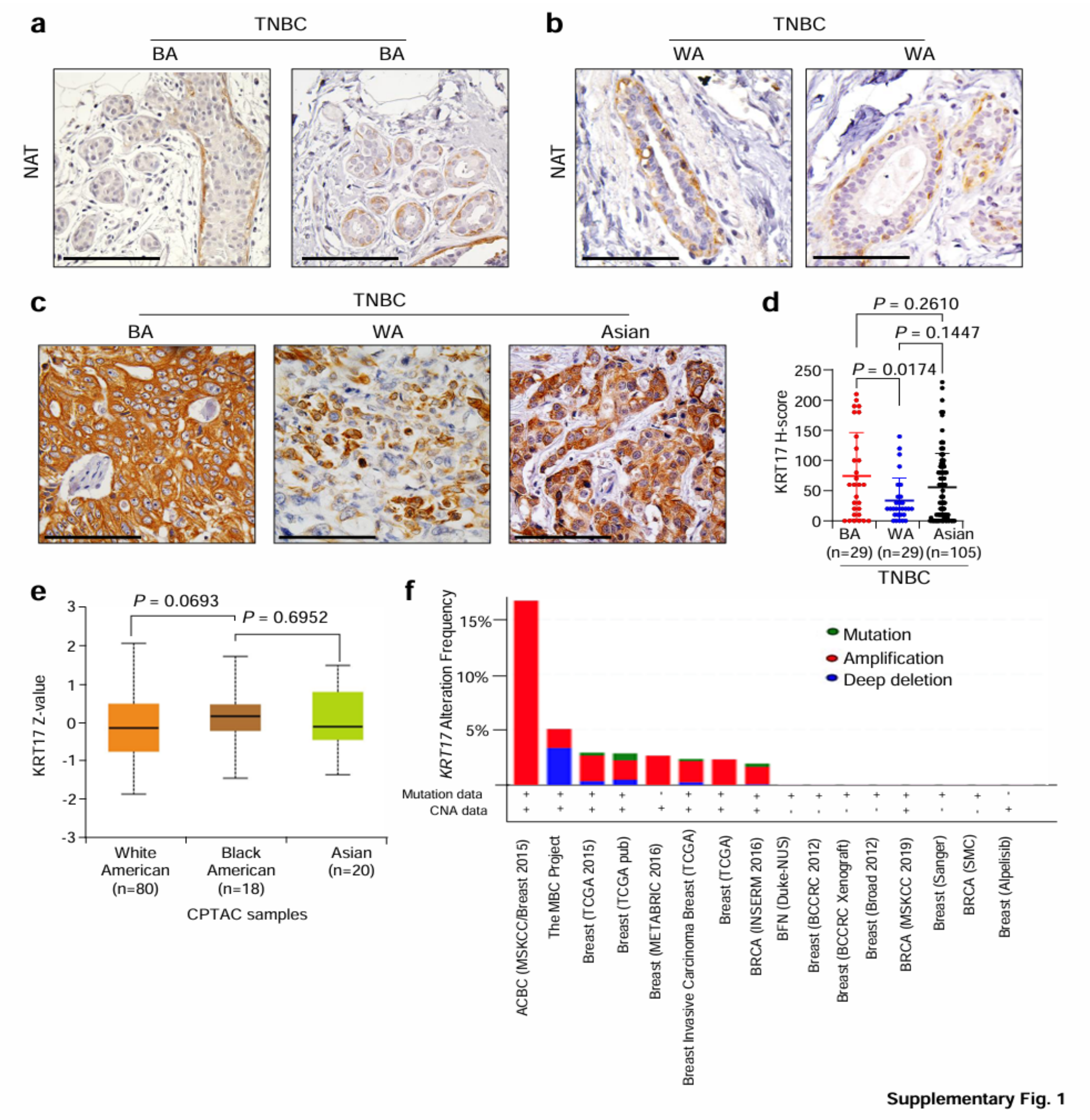

Supplementary fig. 1: KRT17 expression correlates to poor prognosis outcome in BA TNBC. **a** and **b** show KRT17 expression and localization in BA (n=3) and WA (n=3) normal adjacent tissues (NAT). **c** and **d** Representative IHC staining of KRT17 protein expression and quantification in tumors from BA (n = 29), WA (n = 29) and Asian (n= 105) TNBC patient tissues. **e** KRT17 protein expression level on different racial disparity from

UALCAN database. **f** Genomic alteration frequency in the *KRT17* in different breast cancer datasets. Data are presented as the mean ± SEM. Scale bars, 100μm. Statistical significance was determined by one-way ANOVA with Tukey's multiple comparisons test.

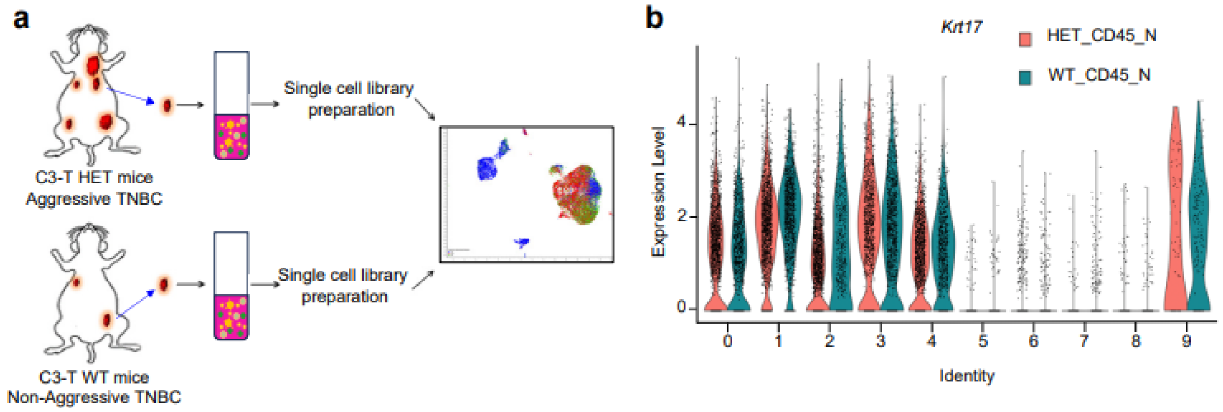

Supplementary Fig. 2

Supplementary fig. 2: Krt17^{high} expression is presented in the sorted CD45^{-} tumor cell clusters by scRNA-seq. a Graphical scheme depicting the scRNA sequencing experiment. b Violin plot showing the *Krt17* gene expression on CD45^{-} (CD45_N) cell cluster from pooled spontaneous C3T-HET and WT tumor.

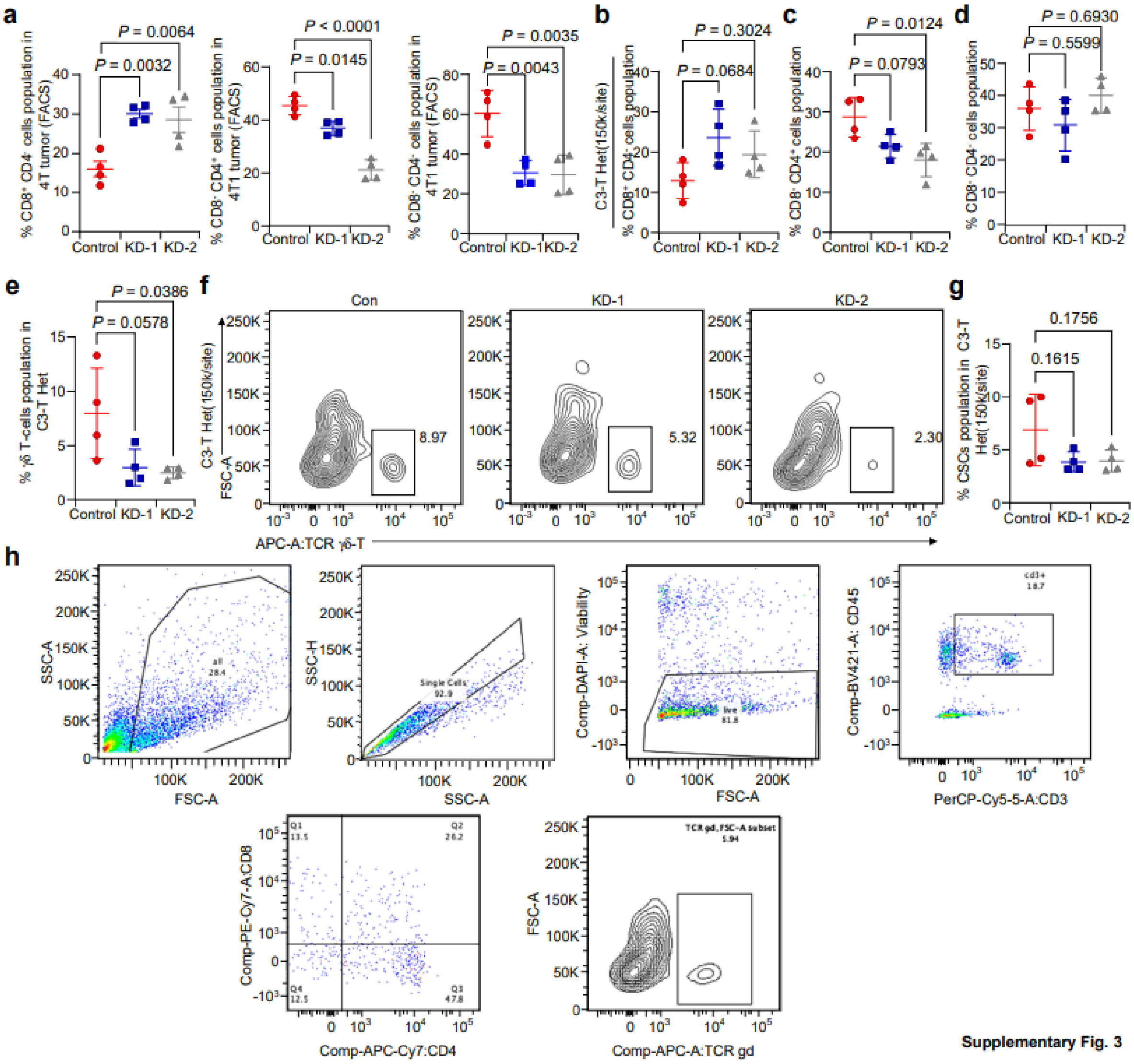

Supplementary Fig. 3

Supplementary fig. 3: Reduced Krt17 is associated with lesser number of γδ T-cells in TME of preclinical TNBC mouse model. a Scatter plot shows the percentage of T-cells population by FACS in different groups of 4T1 tumors, (n=4 tumors/group). **b-d** Scatter plot shows the population of T-cells levels in tumors injected with Epcam$^+$ cells in C3-T HET tumor cells in Rear mice, n=4 tumors/group. **e** Quantification and **f** FACS plots showing γδ T-cells in different tumors of indicated groups. **g** FACS analysis shows the CSCs are reduced in Krt17 KD tumors, n=4 tumors/group. **h** FACS plots showing gates

for $\gamma\delta$ T-cells in C3-T HET TNBC tumor samples. Data was presented as the mean ± SEM. Statistical significance was determined by one-way ANOVA with Sidak's multiple comparisons test.

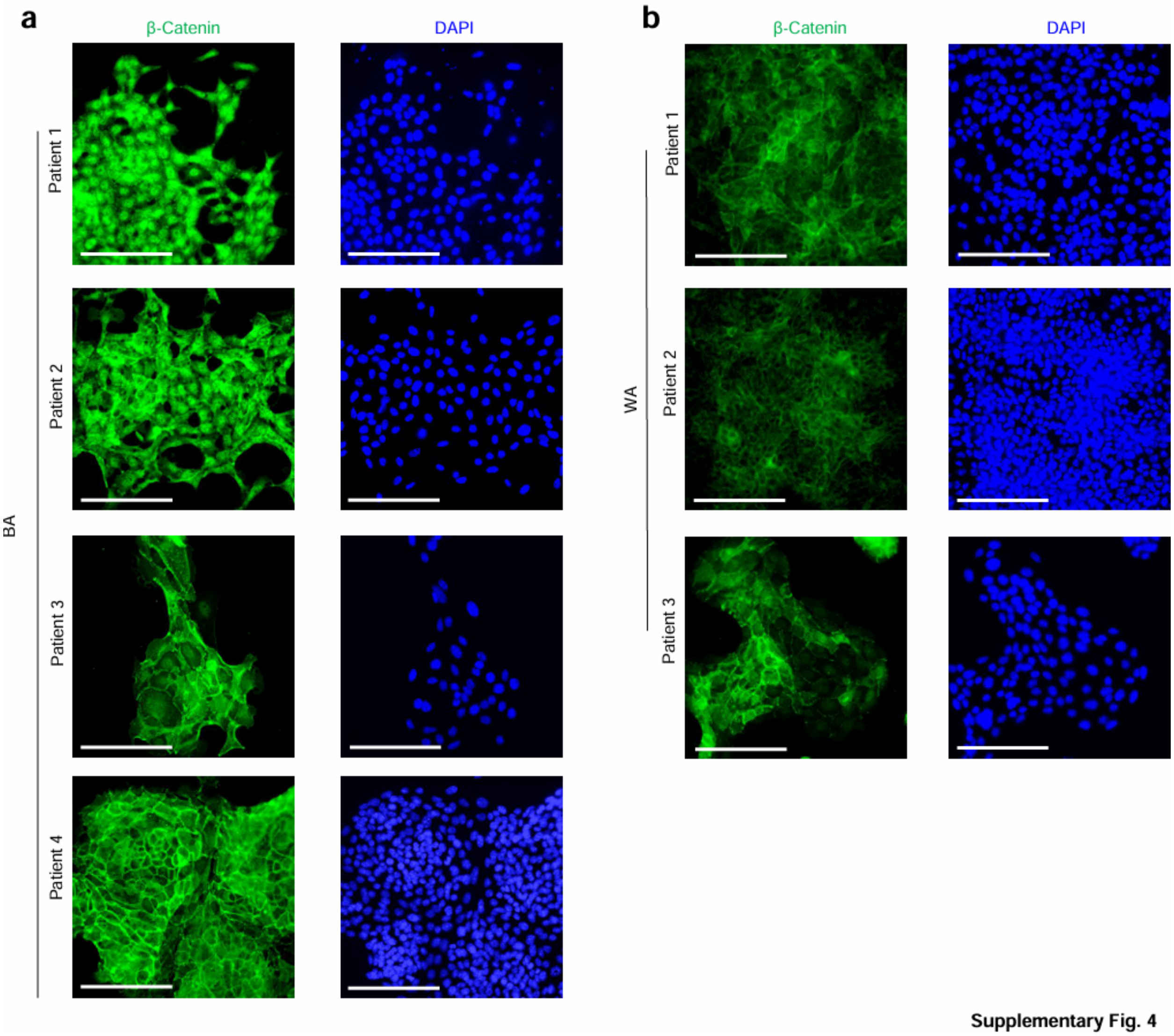

Supplementary fig. 4: BA TNBC patient tissue derived monolayer cells showed higher nuclear expression of β-CATENIN. a Split images of IF for β-CATENIN in BA (n=4) TNBC derived monolayer cells. **b** Split images of IF for β-CATENIN in WA (n=3) TNBC derived monolayer cells. Cell nucleus was counter stained with DAPI (blue) (n=6 FOV/samples with 2 independent biological experiments). Scale bars, 100μm.

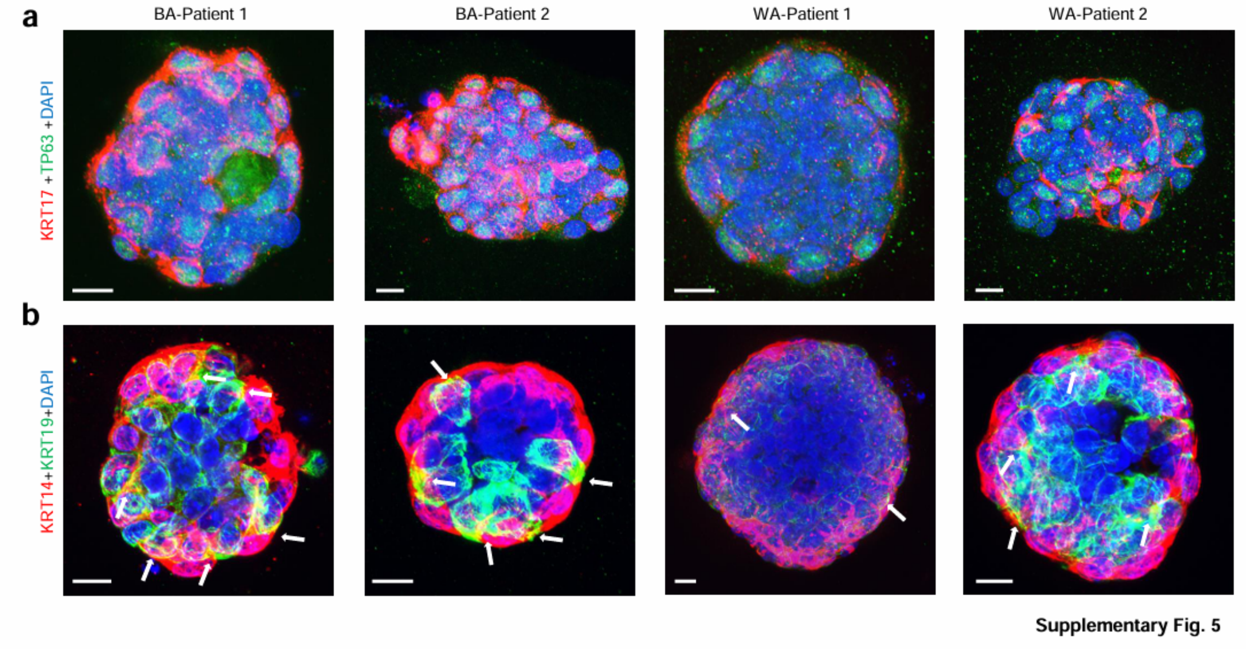

Supplementary fig. 5: BA TNBC patient derived organoids have higher expression of KRT17 and stem cells characteristics. a Confocal image shows KRT17 and TP63 protein expression in patients derived organoids, 2 BA and 2 WA TNBC patients' samples were used (n=2 independent experiments were carried out, with 3 FOV/patient organoids). **b** Expression of Krt14 and Krt19 shows the bipotential stem cells (yellow cells depicted with white arrows) characteristics in BA and WA organoids, 2 BA and 2 WA TNBC patients (Scale bars 10 μm).

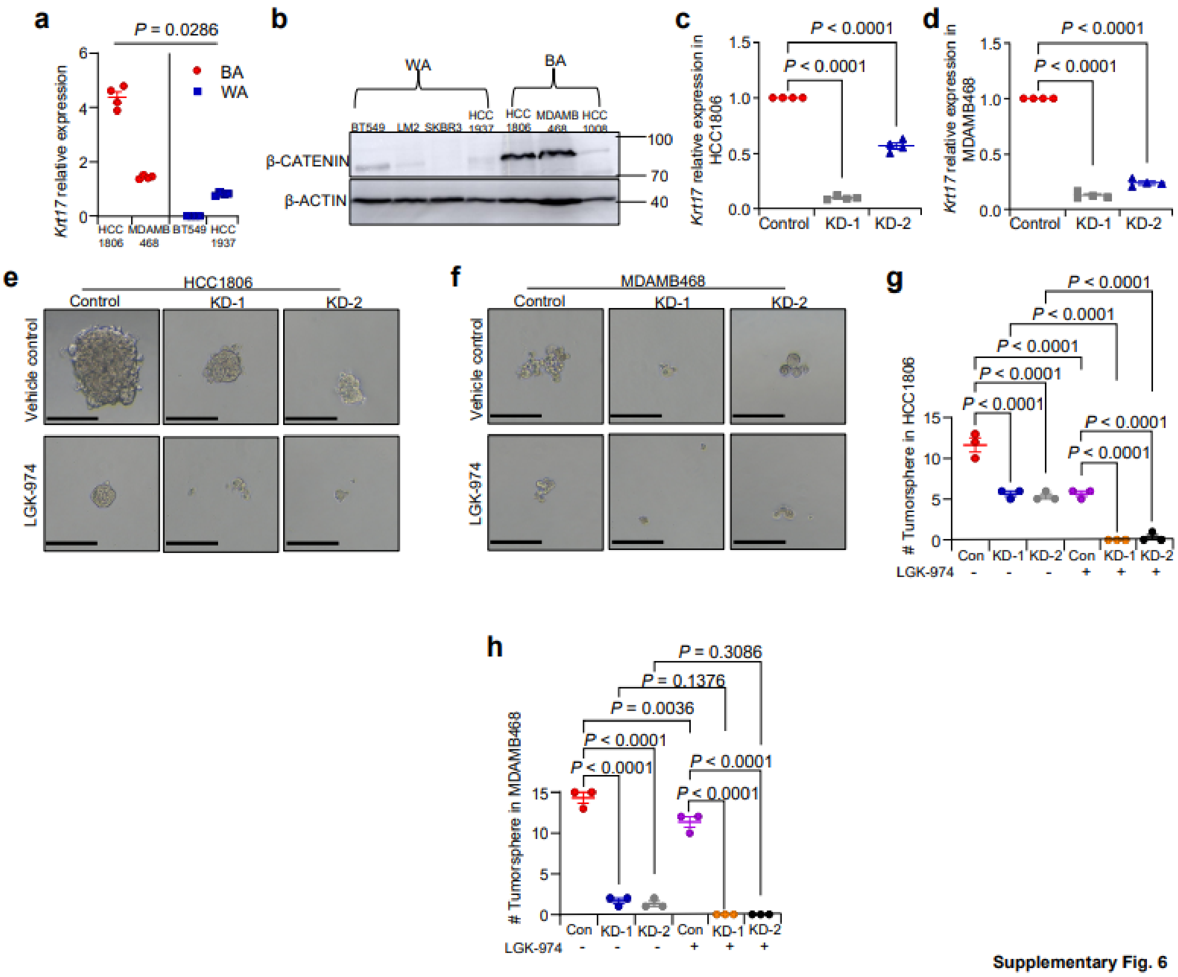

Supplementary Fig. 6

Supplementary fig. 6: BA TNBC human cells have higher expression of KRT17 and β-CATENIN. a Expression of KRT17 in BA and WA TNBC cell lines, *KRT17* expression values were normalized with *GAPDH*. Statistical significance was determined by the Mann-Whitney test. **b** Western blot shows the expression β-CATENIN levels in TNBC BA and WA cells (n=2 independent experiments). **c** and **d** qRT-PCR shows reduced *KRT17* mRNA levels in KD of Krt17 HCC1806 and MDAMB468 cells. Two biological samples were used with technical duplicates, *KRT17* expression values were normalized with *GAPDH*. The data are presented as means ± SEM. Scale bars, 100 μm. **e and f** show the tumorsphere forming ability with or without Wnt inhibitor LGK-974 in Krt17-KD BA

TNBC human cells. **g** and **h** show the number of tumorspheres in indicated cells from **e and f** respectively.

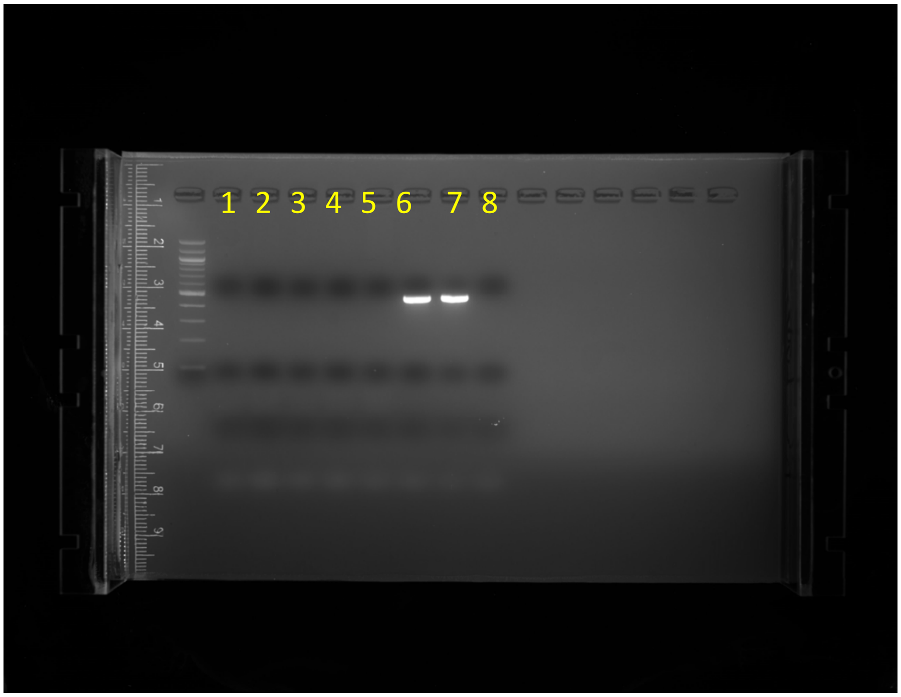

Supplementary fig. 7: ATCC kit: Mycoplasma contamination detection showing no contamination. Lane 1: HCC1806, Lane 2: MDAMB468, Lane 3: BT549, Lane 4: HCC1937, Lane 5: 4T1/LIG, Lane 6: Positive control Lane 7: Positive control-1 Lane 8: Negative control

Original blots of fig. 7i

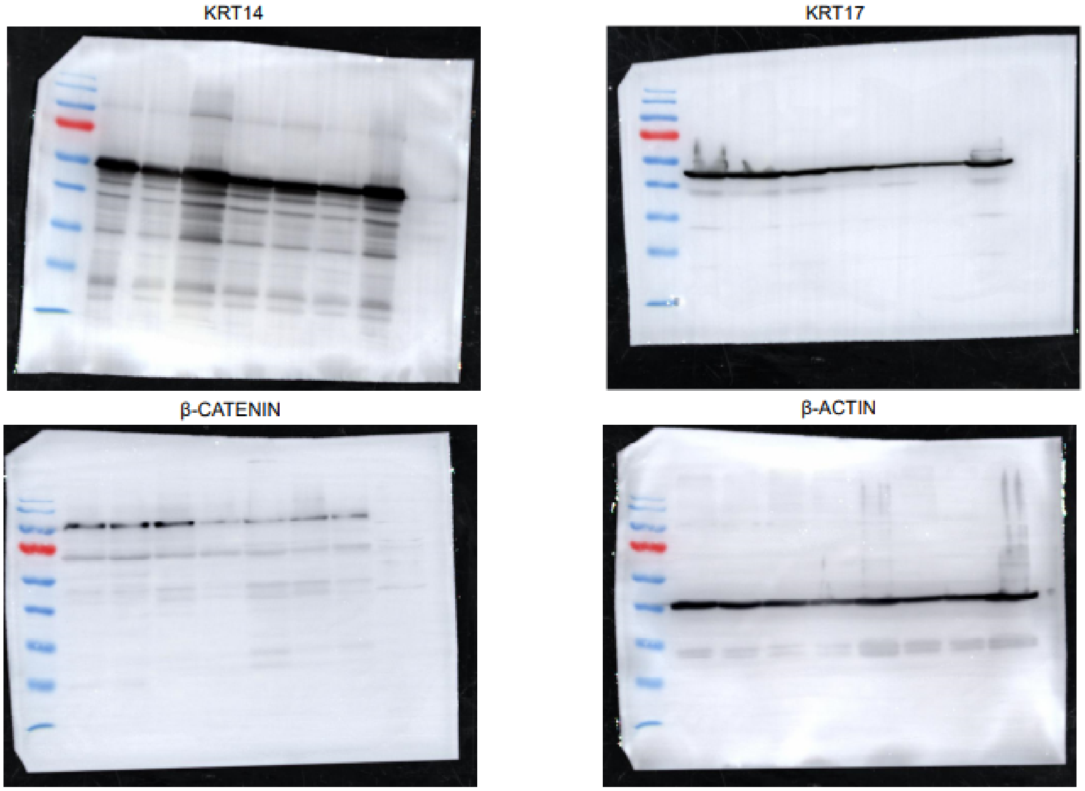

Supplementary Fig. 8

Supplementary fig. 8: Western blot original blots for fig 7.

Original blots of fig. 8a

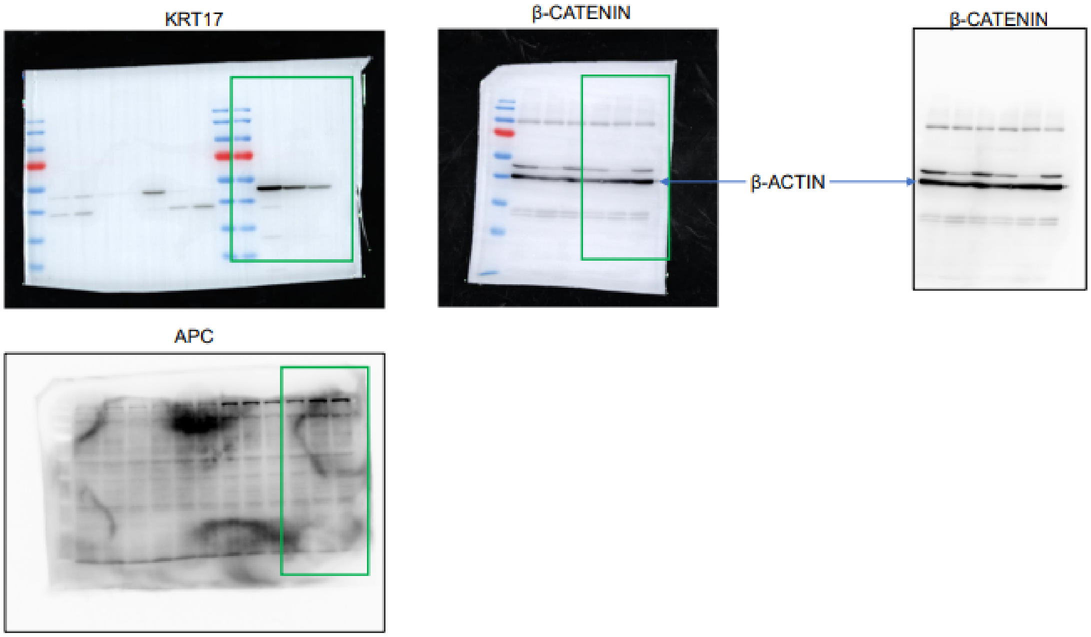

Original blots of supplementary fig. 6b

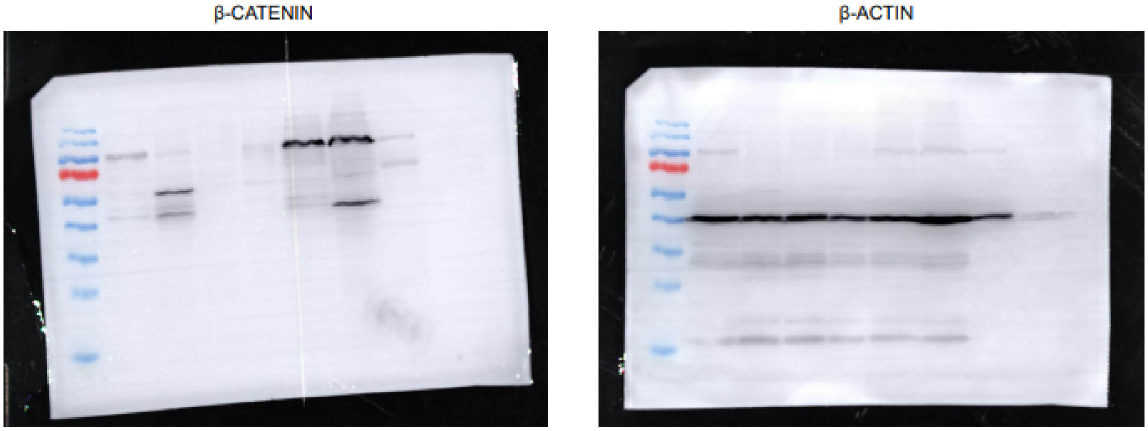

Supplementary Fig. 9

Supplementary fig. 9: Western blot original blots for fig 8.

Supplementary Tables

Supplementary table 1 (Fig. 1h and i: TNBC and Non-TNBC - CHTN patient's samples)

Patient ID	Gender	TNBC/non-TNBC
69710T	F	TNBC
64006	F	TNBC
90357T012	F	TNBC
69918T001	F	TNBC
64002T001	F	TNBC
90359T006	F	TNBC
90355T004	F	TNBC
90356T004	F	TNBC
90352T007	F	TNBC
90354T00	F	TNBC
90351T007	F	TNBC
90358T005	F	TNBC
67190T	F	TNBC
69221	F	TNBC
56393T007	F	TNBC
90360T004	F	TNBC
75336T	F	TNBC
67710T	F	Non-TNBC
685101	F	Non-TNBC
65729T	F	Non-TNBC
67316T	F	Non-TNBC
90369T009	F	Non-TNBC
90361T006	F	Non-TNBC
90368T008	F	Non-TNBC
90366T011	F	Non-TNBC
90363T009	F	Non-TNBC
90367T010	F	Non-TNBC
90370T006	F	Non-TNBC
90365T009	F	Non-TNBC
90362T006	F	Non-TNBC
64973T004	F	Non-TNBC
75240T	F	Non-TNBC
67551	F	Non-TNBC
56455	F	Non-TNBC
51062T005	F	Non-TNBC
65447T001	F	Non-TNBC
90364T006	F	Non-TNBC
57113	F	Non-TNBC

Supplementary table 2 (Fig. 1j and k: Good and Poor survival – University of North Carolina Medical center patient's samples)

**Patient ID**	**Gender**	**TNBC/non-TNBC**	**Good/ Poor survival**
040415-Z	F	TNBC	Good
04-0371B	F	TNBC	Good
03-0408B	F	TNBC	Good
010208B	F	TNBC	Good
08-0206B	F	TNBC	Good
010353-Z	F	TNBC	Good
010049-Z	F	TNBC	Good
03-0078B	F	TNBC	Poor
070331-Z	F	TNBC	Poor
05-0362B	F	TNBC	Poor
101412-Z	F	TNBC	Poor
040593-Z	F	TNBC	Poor
010532-Z	F	TNBC	Poor
140133-1	F	TNBC	Poor
040402-Z	F	TNBC	Poor
99-0141B	F	TNBC	Poor

Supplementary table 3 (Fig. 1l and m: BA and WA – University of Miami patient's samples and Jackson Memorial Hospital)

**Patient ID**	**Gender**	**TNBC/non-TNBC**	**BA/WA**
US14-12123_1	F	TNBC	BA
US15-1832_2	F	TNBC	BA
US15-14677_1	F	TNBC	BA
US14-13755_3	F	TNBC	BA
US18-1157 A1	F	TNBC	BA
US18-12356 A1	F	TNBC	BA
US18-24200 A1	F	TNBC	BA
US18-18310 E7	F	TNBC	BA
US18-21472 A1	F	TNBC	BA
US18-14519A4	F	TNBC	BA
US13-10928 D1	F	TNBC	BA
US13-13032C2	F	TNBC	BA
US14-14971B4	F	TNBC	BA
US14-9226C6	F	TNBC	BA
US14-8697B2	F	TNBC	BA
US22-598 G6	F	TNBC	BA
US16-22209 A1	F	TNBC	BA

US14-7950 A1	F	TNBC	BA
US13-11884_7	F	TNBC	BA
NS24-90_1	F	TNBC	BA
S24-295 F9	F	TNBC	BA
S24-3791 B3	F	TNBC	BA
S24-8637 A1	F	TNBC	BA
S24-11420 A1	F	TNBC	BA
S24-14141 A1	F	TNBC	BA
19-26882	F	TNBC	BA
US20-1442 A6	F	TNBC	BA
19-2800	F	TNBC	BA
S24-15714 D6	F	TNBC	BA
US14-3478A3	F	TNBC	WA
US13-12137_1	F	TNBC	WA
US15-9328 A1	F	TNBC	WA
US14-14207 A2	F	TNBC	WA
US14-6890 C4	F	TNBC	WA
US15-15017_1	F	TNBC	WA
US13-9160 A9	F	TNBC	WA
US18-24764 A1	F	TNBC	WA
US18-7736 A1	F	TNBC	WA
US14-10524 A5	F	TNBC	WA
US18-15171 G5	F	TNBC	WA
US14-18414 B2	F	TNBC	WA
US21-31498	F	TNBC	WA
US21-11217A1	F	TNBC	WA
US21-30497_1	F	TNBC	WA
US21-24789A1	F	TNBC	WA
US23-18657 A4	F	TNBC	WA
US22-5852 B10	F	TNBC	WA
US18-33471 E6	F	TNBC	WA
US24-6501 B1	F	TNBC	WA
Us24-35567 B1	F	TNBC	WA
US13-8499 G3	F	TNBC	WA
US13-12137_1	F	TNBC	WA
US15-21621_1	F	TNBC	WA
US22-5852 B10	F	TNBC	WA
US23-10114 B2	F	TNBC	WA
19-26827	F	TNBC	WA
19-25772	F	TNBC	WA
19-25520	F	TNBC	WA

Supplementary table 4 (Supplementary fig.1a and b BA and WA normal adjacent tissues)

Patient ID	Gender	TNBC/non-TNBC	BA/WA
US13-10928 D1	F	TNBC	BA
US14-7950 A1	F	TNBC	BA
US13-11884_7	F	TNBC	BA
US13-12137_1	F	TNBC	WA
US13-8499 G3	F	TNBC	WA
US15-21621_1	F	TNBC	WA

Supplementary table 5 (Fig. 7a and b: Wnt downstream mRNA gene expression)

Patient ID	Gender	TNBC/non-TNBC	BA/WA	Tumor/Blood
BSSR 2402	F	TNBC	BA	Tumor
BSSR 2813	F	TNBC	BA	Tumor
BSSR 5543	F	TNBC	BA	Tumor
BSSR 2750	F	TNBC	BA	Tumor
BSSR 2601	F	TNBC	BA	Tumor
BSSR 3692	F	TNBC	WA	Tumor
BSSR 4069	F	TNBC	WA	Tumor
BSSR 4117	F	TNBC	WA	Tumor
BSSR 5601	F	TNBC	WA	Tumor
BSSR 4920	F	TNBC	WA	Tumor

Supplementary table 6 (Fig. 7c-i: Patients derived primary cells and organoids establishment)

Patients (BA/WA)	Patient ID	Gender	TNBC/non-TNBC
BA Patient-1	BSSR 2402	F	TNBC
BA Patient-2	BSSR 2813	F	TNBC
BA Patient-3	BSSR 5543	F	TNBC
BA Patient-4	BSSR 5020	F	TNBC
WA Patient-1	BSSR 3692	F	TNBC
WA Patient-2	BSSR 4069	F	TNBC
WA Patient-3	BSSR 5204	F	TNBC

Supplementary table 7 (Fig. 7j: $\gamma\delta$ T-cells analysis in BA and WA TNBC patient's tumor samples by FACS)

**Patient ID**	**Gender**	**TNBC/non-TNBC**	**BA/WA**	**Tumor/Blood**
BSSR 3490	F	TNBC	BA	Tumor
BSSR 2665	F	TNBC	BA	Tumor
BSSR 5543	F	TNBC	BA	Tumor
BSSR 2601	F	TNBC	BA	Tumor
BSSR 2750	F	TNBC	BA	Tumor
BSSR 5020	F	TNBC	BA	Tumor
BSSR 3514	F	TNBC	WA	Tumor
BSSR 3787	F	TNBC	WA	Tumor
BSSR 4117	F	TNBC	WA	Tumor
BSSR 3516	F	TNBC	WA	Tumor
BSSR 6297	F	TNBC	WA	Tumor
BSSR 4920	F	TNBC	WA	Tumor
BSSR 6084	F	TNBC	WA	Tumor

Supplementary table 8 (Fig. 7k: $\gamma\delta$ T-cells analysis in BA and WA TNBC patient's blood samples by FACS)

**Patient ID**	**Gender**	**TNBC/non-TNBC**	**BA/WA**	**Tumor/Blood**
BSSR-2750	F	TNBC	BA	Blood
BSSR-2813	F	TNBC	BA	Blood
BSSR-2953	F	TNBC	BA	Blood
BSSR-4243	F	TNBC	BA	Blood
BSSR 2943	F	TNBC	BA	Blood
BSSR 6046	F	TNBC	BA	Blood
BSSR-3514	F	TNBC	WA	Blood
BSSR-3520	F	TNBC	WA	Blood
BSSR-3828	F	TNBC	WA	Blood
BSSR-4150	F	TNBC	WA	Blood
BSSR 3939	F	TNBC	WA	Blood
BSSR 5222	F	TNBC	WA	Blood
BSSR 5358	F	TNBC	WA	Blood
BSSR 5548	F	TNBC	WA	Blood
BSSR 6084	F	TNBC	WA	Blood
BSSR 5685	F	TNBC	WA	Blood

Supplementary table 9 (Antibodies used for IHC, IF, Western blot and blocking or depletion experiments)

Antibodies	IF/IHC/Western blot (WB) dilution	Blocking	Company and catalogue number
Krt17 (Human-IHC, IF)	1:50	-	Abcam, Cat# ab109725
Krt17 (Mouse-IF)	1:50	-	Cell signaling technology Cat# 4543
CD4 (Mouse-IHC)	1:25	-	Cell signaling technology Cat# 25229
CD8-α (Mouse-IHC)	1:25	-	Cell signaling technology Cat# 98941
Krt14 (Mouse-IF)	1:75	-	Abcam, Cat# ab53115
Krt17 (Confocal)	1:25	-	Abcam, Cat# ab109725
Tp63 (Confocal)	1:25	-	Abcam, Cat# ab735 (4A4)
Krt14 (Confocal)	1:50	-	Abcam, Cat# ab53115
Krt19 (Confocal)	1:25	-	DHSB, Cat# AB 3133570
β-Catenin (IF)	1:25	-	Invitrogen, Cat# 138400
Krt17 (Human-WB)	1:1000	-	Abcam, Cat# ab109725
Krt14 (WB)	1:1000	-	Abcam, Cat# ab53115
β-Catenin (WB)	1:1000	-	Invitrogen, Cat# 138400
APC (WB)	1:1000	-	Abcam, Cat# ab40778
β-Actin (WB)	1:20000	-	Sigma-Aldrich Cat# A5441
IgG control	-	100ug/mouse	BioXcell, Cat # BE0089
CD8a	-	100ug/mouse	BioXcell, Cat# BE0061
TCR γδ	-	250ug/mouse	BioXcell, Cat# BE0070

Supplementary table 10 (FACS antibodies)

Antibodies	Flurochrome	Dilution	Company and catalogue number
CD45	FITC	1:50	Biolegend Cat# 103108
CD4	APC Cy7	1:50	BD Biosciences Cat# 552051
CD8a	PE Cy7	1:50	BD Biosciences Cat# 552877
CD3	PerCP/Cyanine 5.5	1:25	Biolegend Cat# 100218
TCR γδ	APC	1:50	Biolegend Cat# 118116
CD24	PE	1:50	BD Biosciences Cat# 553262
CD44	APC	1:50	BD Biosciences Cat# 559250
CD45	BV421	1:50	BD Biosciences Cat# 563890
CD45 (Human)	APC/Cyanine7	1:50	Biolegend Cat# 301014
CD3 (Human)	BV605	1:50	Biolegend Cat# 317322
CD8 (Human)	PE Cy7	1:50	Biolegend Cat# 344711
CD4 (Human)	PerCP/Cyanine 5.5	1:50	Biolegend Cat# 317428
TCR γδ (Human)	APC	1:50	Biolegend Cat# 331212

Supplementary table 11 (Primers used in quantitative PCR and for genotype)

Gene	Forward (5’→3’)	Reverse (5’→3’)
mKrt17	CCTGACTCAGTACAAGCCAAA	CTGTTCCCGGGATGAGATG
mLef1	GCCACGGATGAGATGATCCC	TTGATGTCGGCTAAGTCGCC
mTcf1	ACAGTGCTCTAGGCTGTCC	CCGGCTCTTTCAGAATGGGT
mAscl2	TGAGGTCCACCAGGAGTCAC	CAGGAGCTGCTTGACTTTTCC
mGapdh	TTCCACTCTTCCACCTTCGATGC	GGGTCTGGGATGGAAATTGTGAGG
hKRT17	ATCCTCAACGAGATGCGTGA	CTCGCGGTTCAGTTCCTCT
hLEF1	CTATCCCAATGGCAGAGGTGG	GGGTCCCTTGTTGTAGAGGC
hLBH	GGACGCAGGGACCGTTTTTA	TCTCAGATAGTCGGGGCAGT
hGAPDH	GGAGTCAACGGATTTGGTCGTA	GGCAACAATATCCACTTTACCAGAGT
LacZ Genotype primers	GCCACGTGTATAAGATACACCTGC	GCGCATCGTAACCGTGCATCTGCC
C3-T Genotype primers	CTCCCAACCCCAGAGGTAGT	AGACCCCAGATCCAGAAAGG
	CAGAGCAGAATTGTGGAGTGG	GGACAAACCACAACTAGAATGCAGTG
