# Supplementary material for: KRT17 promotes triple negative breast cancer through activation of Wnt signaling and γδ T-cells recruitment
Source: Commun Biol. 2026 Mar 26;9:676. doi: 10.1038/s42003-026-09897-0 (PMC13183921; doi:10.1038/s42003-026-09897-0)
Supplement: Supplementary file 3 — Description of Additional Supplementary Files [file 42003_2026_9897_MOESM3_ESM.docx]

**Description of Additional Supplementary Files**

**File name:** Supplementary Data

**Description:** Raw data file.
